# Defining myocardial infarction: grades of severity or different aetiology

**DOI:** 10.1093/ehjacc/zuaf025

**Published:** 2025-02-17

**Authors:** Andrew P DeFilippis, Michael J Blaha

**Affiliations:** Department of Medicine, Vanderbilt University Medical Center, 1215 21st Avenue South, MCE 5th Floor, North Tower, Nashville, TN 37232, USA; Ciccarone Center for Prevention of Cardiovascular Disease, Division of Cardiology, Department of Medicine, Johns Hopkins Medical Institutions, Baltimore, MD, USA

Descriptions of acute myocardial infarction (MI) in the medical literature date back to the 19th century, with taxonomy and diagnostic criteria evolving since that time.^1^ One example of this evolution is the discovery and development of assays for detecting cardiac troponin (cTn) allowing for the identification of an increasing number of patients with acute myocardial injury—a necessary but not sufficient finding for the diagnosis of MI. Myocardial injury is a broad diagnostic category defined by an elevated cTn, including MI but also non-MI causes of myocardial injury. Myocardial infarction is a specific type of myocardial injury caused by myocardial ischaemia—insufficient oxygen delivery to the myocardium. Myocardial infarction is defined and categorized in many ways, including size (e.g. large or small), aetiology (e.g. atherothrombotic), electrocardiographic consequences (e.g. ST-segment elevation), histology (e.g. transmural coagulative necrosis), and location (e.g. anterior), and even the lack of findings have been used to define types of MI, e.g. myocardial infarction with non-obstructive coronary arteries.

The most widely utilized definition of myocardial injury and MI, the Fourth Universal Definition of MI (4th UDMI), identifies MI as an acute myocardial injury manifested as dynamic changes in cTn concentration, secondary to ischaemia, defined by a combination of symptoms, ECG (ST-segment changes or the development of pathological Q-waves), coronary angiography findings, or evidence of a new regional wall motion abnormality.^2^ The UDMI further sub-classifies MI with a combination of aetiologic considerations and diagnostic findings. Type 1 MI is a coronary atherothrombotic event, due to coronary atherosclerotic plaque rupture or erosion. Type 2 MI occurs secondary to an acute imbalance in myocardial oxygen supply/demand in the absence of acute coronary atherothrombosis. Acute myocardial injury (rise and/or fall in cTn) in the absence of ischaemia is classified as acute non-ischaemic myocardial injury.

While the UDMI provides aetiological taxonomy, diagnostic criteria for categorizing the types of myocardial injury remains ambiguous for certain patients, particularly when differentiating type 2 MI and acute non-ischaemic myocardial injury.^3,4^ Challenges to establishing clear diagnostic criteria include the fact that both diagnoses encompass multiple precipitating aetiologies. For example, the UDMI defines type 2 MI as an acute ischaemic myocardial injury not secondary to coronary plaque disruption and atherothrombosis. This includes coronary causes like spasms and embolic occlusion, cardiac causes like tachyarrhythmias, or systemic drivers like hypoxaemia. When evaluated as a singular entity, type 2 MI is associated with high morbidity and mortality; however, no specific therapy has been identified to effectively treat or prevent this condition.^3,5^ To improve outcomes for this common and deadly diagnosis, clear, widely available, and economically feasible consensus diagnostic criteria for type 2 MI are needed to foster effective therapeutic research.^6^ In this regard, we applaud Boeddinghaus *et al.*^7^ for their examination of the impact of additional testing and attempted re-categorization of patients that satisfy the current diagnostic standard for type 2 MI, as defined by the 4th UDMI. By default, adding additional criteria to make a diagnosis will reduce the number of patients that meet diagnostic criteria.

Inclusion criteria required study participants to meet UDMI criteria for type 2 MI. In addition, this study required the examination of coronary anatomy via computed topography or invasive angiography with optical coherence tomography of coronary lesions when discovered, and examination of the myocardium with cardiac magnetic resonance imaging (MRI) or transthoracic echocardiography when cardiac MRI was not feasible (not all patients received the same tests). After receiving the required studies, participants were categorized as *spontaneous MI*, defined as confirmed coronary atherothrombosis, or spontaneous coronary dissection, embolism, vasospasm, in-stent restenosis, late stent thrombosis, and late graft failure, or as *secondary MI*, defined as the presence of obstructive coronary disease (>50% left main or >70% a major epicardial vessel stenosis), functional myocardial impairment (regional wall motion abnormality in a coronary distribution), or scarring (infarct pattern late gadolinium enhancement). Participants who failed to meet the criteria for these two categories were categorized as having *no MI*, akin to the UDMI category of acute non-ischaemic myocardial injury since all participants had a rise and/or fall in cTn to meet the study inclusion criteria of type 2 MI.

This study found that among 100 participants who on upfront screening fulfilled UDMI criteria for type 2 MI and the required study testing, 25% were newly classified as *spontaneous MI*, 31% were classified as *secondary MI*, and 44% were classified as *no MI*. The further classification of participants, all of which initially meet UDMI for type 2 MI, produced groups with clinically meaningful differences that may provide insight into therapeutic strategies. Participants categorized as *secondary MI* had similar rates of chest pain and ischaemic EKG findings on presentation compared to those categorized as *no MI* but a higher rate of death, subsequent MI, or hospitalization with heart failure [55% (17/31) vs. 16% (7/44), *P* < 0.001] over a median follow-up of 4.4 years. These findings suggest that the additional testing required in this trial identified a subgroup of patients that meet UDMI criteria for type 2 MI, not otherwise identified with typical diagnostic criteria of chest pain and ischaemic EKG findings, that are at greater risk for major adverse cardiac events.

Does this study demonstrate the efficacy of a new classification system that requires extensive additional testing, which will have significant implications on financial resources including the hospital time it takes to complete a work-up? In participants reclassified as *spontaneous MI*, five had acute atherothrombosis; therefore, per UDMI criteria, these participants were initially misclassified as type 2 MI and it was the additional testing, not a new classification system, that identified the atherothrombotic (type 1) MI. The remaining participants in the *spontaneous MI* category continue to satisfy UDMI criteria for a type 2 MI: eight had a coronary embolism (*n* = 8), seven had a spontaneous coronary dissection (*n* = 7), and five were diagnosed with coronary vasospasm (*n* = 5). Thirty-one per cent of the study participants were reclassified as *secondary MI*; all of which satisfy UDMI criteria for type 2 MI, but essentially with confirmed severe coronary disease.

Do differences in outcomes between these new subcategories of patients who all meet UDMI criteria for type 2 MI represent differences in pathology or differences in severity of the same pathology? Older age, comorbid conditions, higher creatinine, and lower haemoglobin were all more prevalent in the *secondary* vs. *no MI* and, therefore, may explain the higher incidence of clinical cardiovascular outcomes for participants diagnosed with *secondary MI*. The lower presence of ischaemia via electrocardiogram, cTn, and myocardial scar identified by cMRI in the *secondary* vs. *spontaneous MI* but greater clinical cardiovascular event risk in this study would suggest that factors other than the extent of myocardial injury are responsible for differences in cardiovascular outcomes.

Is the presence of obstructive coronary artery disease (CAD), as defined by Boeddinghaus *et al.* (>50% left main or >70% a major epicardial vessel stenosis), useful in defining a specific subtype of patients that meets criteria for UDMI type 2 MI? Other studies have already demonstrated a higher incidence of cardiovascular morbidity and mortality among patients who have acute myocardial injury and CAD as compared to myocardial injury without concomitant CAD.^8^ Further, the association of CAD with greater incident cardiovascular morbidity and mortality is not limited to patients with acute myocardial injury. In fact, the presence of CAD is associated with unfavourable outcomes in most any patient population, including asymptomatic primary prevention patients. While we believe that it is imperative to address CAD in acute myocardial injury, this is also true in patients without acute myocardial injury. Coronary artery disease is complex beyond what is represented by stenosis alone; additional research is needed to determine if and what characteristics of CAD are most important in the categorization, outcome, and therapeutic approach to patients with acute myocardial injury.

In addition to identifying relevant differences in pathology, for a new classification system to be effective, its use must be reproducible. How often do physicians agree on the classification of any one patient with this proposed classification system? Data on the agreement between adjudication physicians in this study was not presented and would be a welcomed addition to this work. Ultimately, the performance of the classification system among physicians with patients independent of this study will be needed to predict performance in clinical practice.

We agree with the authors’ stated goal of adjusting MI taxonomy to encourage more accurate and widespread utilization. Achieving such a goal would foster robust research leading to specific therapies for different myocardial injury patients.^9^ The categorization utilized in this study for patients who meet the criteria for a UDMI type 2 MI is part of a comprehensive MI categorization system proposed by the same research group.^10^ The proposed MI classification system starts with defining the patient population of interest, ‘Symptoms or signs giving suspicion of myocardial ischaemia with a dynamic elevation in cardiac troponin’, and further divides MI by clinical setting (spontaneous, secondary, or procedural) and then uses specific cardiac imaging findings to define five diagnostic categories: spontaneous MI, spontaneous MI due to a specified coronary mechanism other than atherothrombosis, secondary MI, procedural MI, and acute myocardial injury not due to MI. We applaud this creation and evaluation of an alternative myocardial injury taxonomy by Boeddinghaus *et al.* However, we caution that myocardial injury taxonomy should allow for clarity in aetiology and a parallel space to define severity—both should dictate diagnostic and therapeutic strategy. The lack of CAD, a new regional wall motion abnormality, or scar identified by cMRI in the setting of a rising cTn does not always determine aetiology and may inappropriately miss the presence of ischaemic myocardial events that are below the threshold of detection via echocardiography or cMRI. These criteria may be more informative of the severity vs. the aetiology of the myocardial injury events.

We agree that the severity of the myocardial injury is essential in balancing the risks and benefits of diagnostic and therapeutic interventions, but as the risks of these diagnostic and therapeutic interventions change, so should implementation thresholds. Reserving the designation of MI only for patients with an acute ischaemic insult resulting in a regional wall motion abnormality on echocardiography or some other measure of severity may delay the implementation of new therapeutics for which the risk–benefit calculation is more favourable at a lower level of disease severity. Therefore, while limiting the designation of MI for patients with clearly established coronary disease or myocardial impairment may be successful in pulling out ‘more severe traditional type 2 MIs’, the implications for acute myocardial injury events with lesser severity may become even more unclear. In summary, more work is needed to determine if the proposed increase in diagnostic testing to further categorize patients with acute ischaemic myocardial injury will be worth the squeeze.

## Data Availability

No new data were generated or analysed in support of this research.
